# The Heck Reaction Applied to 1,3- and 1,2-Unsaturated Derivatives, a Way towards Molecular Complexity

**DOI:** 10.3390/molecules15042667

**Published:** 2010-04-13

**Authors:** Annamaria Deagostino, Cristina Prandi, Silvia Tabasso, Paolo Venturello

**Affiliations:** Dipartimento di Chimica Generale e Chimica Organica, Università di Torino, Via Pietro Giuria, 7, I 10125, Torino, Italy

**Keywords:** Mizoroki–Heck reaction, palladium, 1,2-dienes, 1,3-dienes

## Abstract

This review is an overview of the last ten years’ use of the Mizoroki–Heck coupling applied to 1,2- and 1,3-dienes. Since both these systems form π-allyl palladium intermediates in Pd(0) coupling, they show particular chemical behavior. Many examples of 1,2-dienes Heck reactions are presented. 1,2-Dienes are important substrates because of their high reactivity that makes them useful building blocks for the synthesis of biologically relevant structures.

## 1. Introduction

1,2-Dienes and 1,3-dienes are interesting substrates among the unsaturated systems because of their high reactivity. In fact, when they undergo a carbopalladation process a π-allyl palladium intermediate is formed and at least two reactive pathways have to be considered. If a nucleophile is present in the reaction medium the addition product is obtained, otherwise a β-H elimination occurs [1]. 

**Scheme 1:**
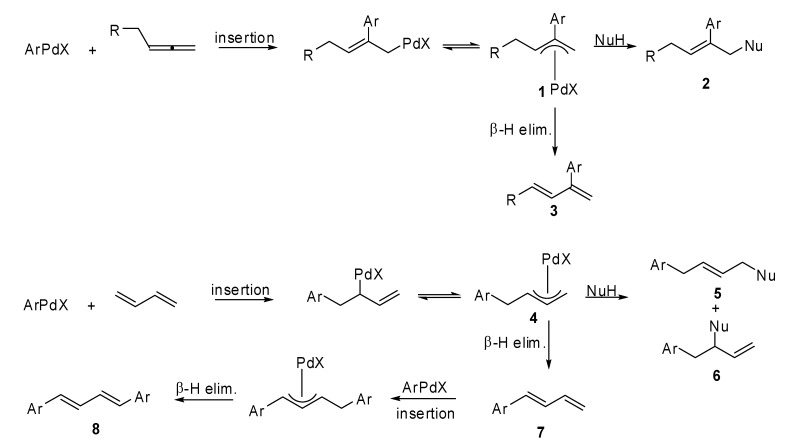
General reactivity of 1,2- and 1,3-dienes in Mizoroki–Heck reaction.

As described in Scheme 1, allenes easily undergo carbopalladation and for this reason have been widely exploited in organic synthesis as useful intermediates for biologically relevant compounds. In this case, the aromatic group attacks the central sp carbon of the allenic moiety creating the corresponding π-allyl palladium intermediate **1**, and then the nucleophile attacks the less hindered terminus of complex **1** according to a 1,2-addition reaction, affording product **2**. In the case in which the termination step is a β-H elimination, 2-substituted-1,3-dienes **3** are achieved.

When conjugated dienes are involved in the Pd(0) coupling, both the 1,4- and 1,2-addition products (**5** and **6** in Scheme 1) are produced as a consequence of the nucleophile entry, whereas the corresponding substituted 1,3-diene **7** is obtained as the result of an elimination process. Sometimes diene **7** can react once more with the aryl halide producing the 1,4-diarylated diene **8**, according to a β-H elimination process on the newly formed π-allyl palladium complex.

The general reactivity of conjugated dienes and the direct formation of their palladium π-allyl complexes was already described by Heck [2]. The Grigg research group studied the palladium catalyzed tandem cyclization anion capture processes. The cases of carbon-, nitrogen- and oxygen- centered nucleophiles were described [3]. They also described the reactivity of alkylallenes in the Heck-Diels-Alder cascade processes [4]. Larock and coworkers greatly contributed to the description of the chemistry of Heck coupling of 1,2- and 1,3-dienes [5,6,7,8]. They first described the carbo- and heteroannulation of dienes. The enantioselective version was also investigated [9]. The intramolecular version of this process has been widely utilized for the synthesis of complex molecules. The Pd catalyzed cyclizations of haloallenes and allenols were studied and described by the Ma research group [10,11].

## 2. 1,3-Dienes

**Scheme 2:**
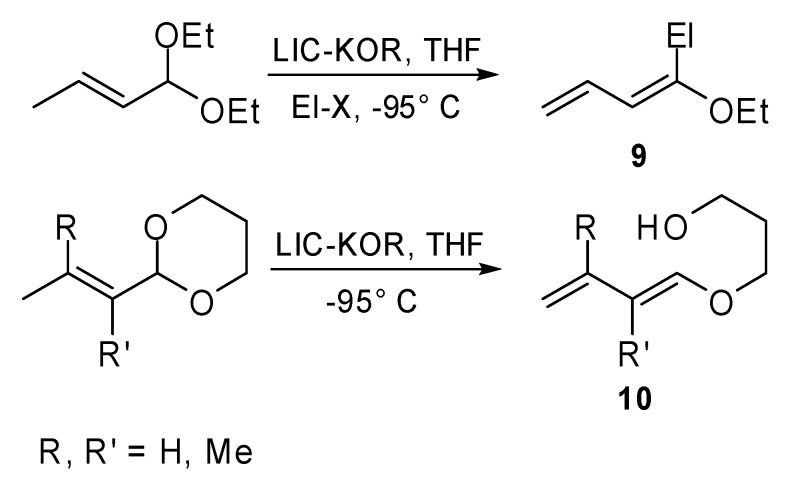
Reaction of α,β-unsaturated acetals with superbase LIC-KOR.

The Pd(0) catalyzed coupling of 1-alkoxy-1,3-dienes, obtained by the reaction of α,β-unsaturated acetals with superbase LIC-KOR (equimolar mixture of *n*-butyllithium and potassium *tert*-butoxide) in the presence of the suitable electrophile (Scheme 2), has been studied in our laboratory [12]. 

**Scheme 3:**
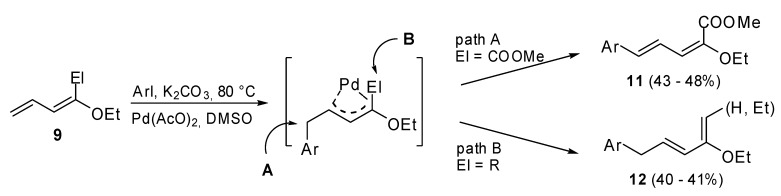
Heck reaction on 1-ethoxy-buta-1,3-dienes.

When derivatives **9** were coupled with aryl halides**, **two different pathways (Scheme 3) were observed. The choice of pathway depends on the group bonded to the C^1^ of the dienic moiety. In the case of *(E)*-methyl 2-ethoxypenta-2,4-dienoate (El = COOMe), the corresponding 4-aryl derivatives **11** were isolated in a regio- and stereoselective manner (path A, Scheme 3). On the contrary, in the case of 1-alkyl-1-alkoxydienes (El = Me, *n*-Pr) arylated dienes **12**, that are isomers of the expected dienes, were synthesized (path B, Scheme 3). A common π-allylpalladium intermediate which undergoes a β-hydride elimination process from two different sites was hypothesized. Pathway B was probably favored for steric reasons.

**Scheme 4:**
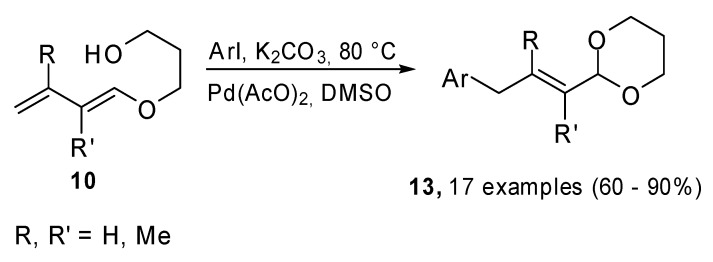
Heck reaction on 1-(3-hydropropoxy)buta-1,3-dienes with aryl iodides.

When the Heck cross coupling was carried out on compounds **10**, the reaction followed a different pathway because of the presence of the nucleophilic hydroxyl group in the substrate (Scheme 4). The 1,3-dioxane ring was reformed and the process formally led to a γ-arylation process on the α,β-unsaturated protected carbonyl compounds.

This methodology was extended to arendiazonium salts, because the Heck reaction on these substrates is mild and fast. Moreover, their use is synthetically convenient in comparison to aryl halides since many of them, especially iodides, are prepared from diazonium salts [13]. The couplings were carried out in anhydrous MeCN at room temperature in the presence of NaAcO as the base and Pd(AcO)_2_ as the catalyst. The reactions showed total regio- and stereoselectivity, unfortunately the coupling yields were modest, probably they were affected by the stability of either the diazonium salt or the dienes under the reaction conditions.

**Scheme 5:**
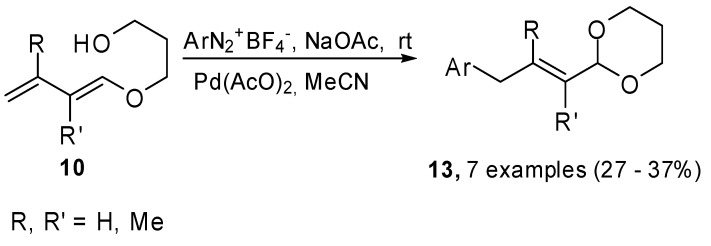
Heck reaction on 1-(3-hydropropoxy)buta-1,3-dienes with arendiazonium salts.

**Scheme 6:**
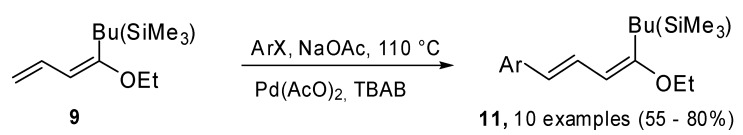
Heck reaction on 1-ethoxy-buta-1,3-dienes in pure TBAB.

**Scheme 7:**
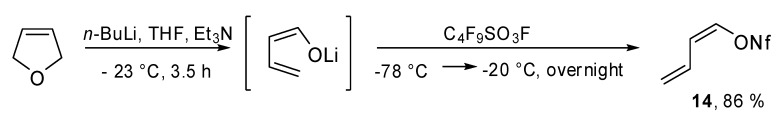
Synthesis of (*Z*)-buta-1,3-dien-1-yl nonaflate by lithiation of 2,5-dihydrofuran.

As the yields of the Heck coupling between 1-ethoxybuta-1,3-dienes **9** and aryl halides described in Scheme 3 were not satisfactory, different reaction conditions were tried in our laboratory. In particular the effect of the addition of ionic liquid to the reactive mixture was evaluated and it was observed that the presence of TBAB increased both the yields and the reaction rates (Scheme 6) [14]. The best results were obtained using a mixture of TBAB and DMSO as the solvent, NaAcO as the base and Pd(AcO)_2_ as the catalyst. However the subsequent couplings were carried out in pure TBAB, as the possibility of developing a solvent free method was, in our opinion, more significant than the longer reaction times it caused (4 h instead of 2 h). Moreover the Heck reaction was successfully applied to aryl bromides.

(*Z*)-Buta-1,3-dien-1-yl nonaflate (**14**) was obtained through aldehyde-free pathways, exploiting the lithiation of 2,5-dihydrofuran followed by the cyclo fragmentation of the metallated heterocycle by Reissig and coworkers (Scheme 7). 

**Scheme 8:**
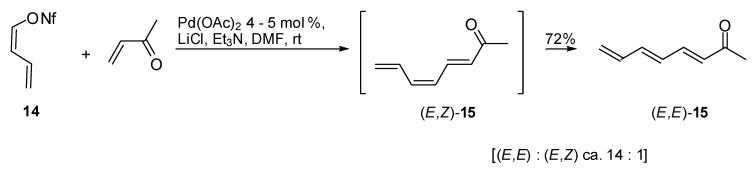
Heck reaction of (*Z*)-buta-1,3-dien-1-yl nonaflate.

The so obtained conjugated diene **14** was found to react with monosubstituted alkenes under phosphine free conditions and using Pd(OAc)_2_ as the catalyst. It was observed that the coupling was influenced by the presence of lithium chloride as the co-catalyst, the product (3*E*, 5*Z*)-octa-3,5,7-trien-2-one (**15**) was obtained in good yields and high (*E*)-selectivity. In fact as expected, nonaflate **14** seemed to react while retaining its configuration as illustrated in Scheme 8 [15]. 

**Scheme 9:**
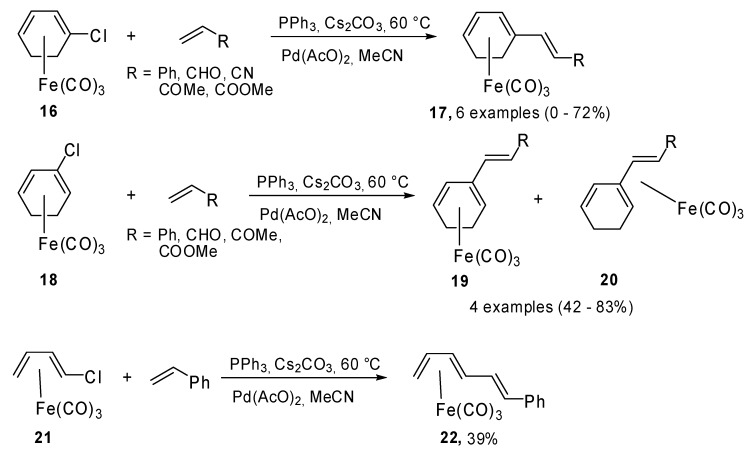
Heck reactions of vinyl chlorides and diene−Fe(CO)_3 _complexes.

Li and coworkers reported an interesting example of Heck coupling between vinyl chlorides **16**, **18** and **21** and different olefins (Scheme 9) [16]. In particular, they demonstrated that the vinylic C-Cl bond is activated upon complexation with Fe(CO)_3_. The diene was firstly and easily bonded to Fe(CO)_3 _and then reacted under the Heck conditions. In consideration of the ready decomplexation of the diene−Fe(CO)_3 _under mild conditions, this method could find many applications in organic synthesis.

## 3. 1,2-Dienes

As previously outlined, allenes are reactive substrates in Pd(0) catalyzed reactions and, in recent years, much work has been done to find new synthetic methods which utilize these molecules for the preparation of structurally complicated products.

**Scheme 10:**
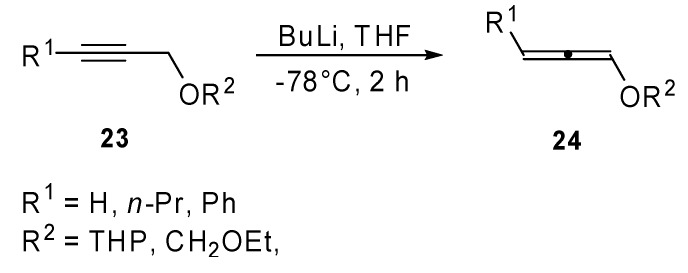
Isomerization reaction of alkynes to allenes in the presence of BuLi.

Recently, in our laboratory some attention has been paid to the reactivity of protected 1,2-dien-1-ols in the Heck reaction conditions. A new synthetic method for the preparation of α-arylated α,β-unsaturated aldehydes (**26**) has been proposed [17]. Protected dienols **24** were prepared from the corresponding alkynes **23** by reaction with butyllithium (Scheme 10). As has been shown in literature, a mixture of propargyl and allenyl ethers was obtained in the case of internal alkynes.

**Scheme 11:**
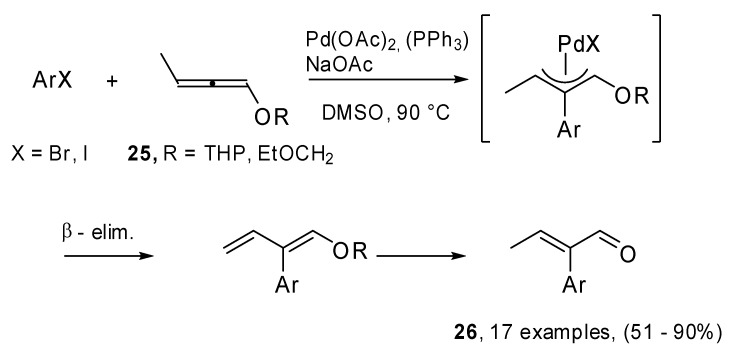
Heck couplings of aryl halides and allenols to afford α-arylated α,β-unsaturated aldehydes.

The methodology was applied to differently substituted iodo- and bromoarenes (Scheme 11), in DMSO at 90 °C. NaAcO was used as the base and PPh_3_ was added in the case of bromo derivatives. Seventeen examples were reported. The reactions were regio- and stereoselective, furthermore they turned out to be strongly influenced by substituent steric effects. Moreover, in the case of bromoarenes electronic effects appeared to be important. 

**Scheme 12:**
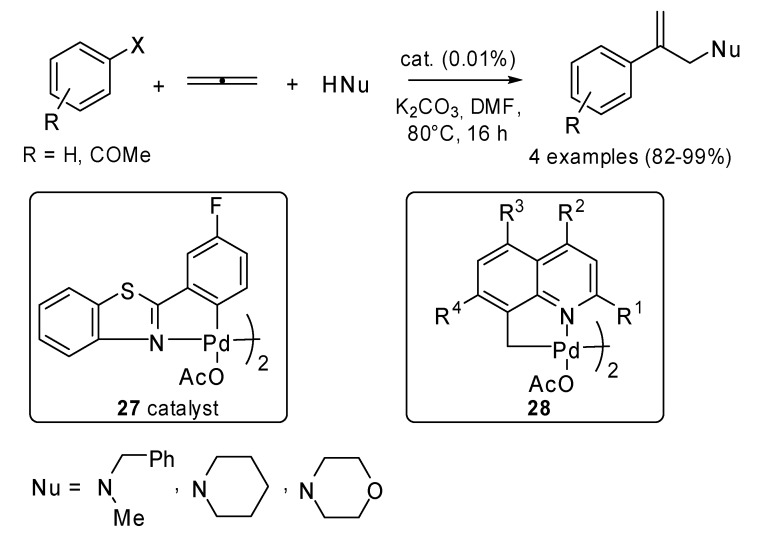
Three-component cascade process involving haloarenes, 1,2-propene and different amines.

Grigg and coworkers did a lot of work in studying the reactivity of allenes in Pd(0) catalyzed reactions. The use of non-phosphine palladacycles **27** as efficient catalysts in Heck reaction was applied to a three-component cascade process involving haloarenes, 1,2-propene and different amines (Scheme 12) [18]. Some non-phosphine 8-methyl quinoline based dimeric palladacycles **28**, which possess an sp_3_–C bond, were tested too. They proved to be efficient pre-catalysts for both Heck and 3-component cascade processes [19].

**Scheme 13:**
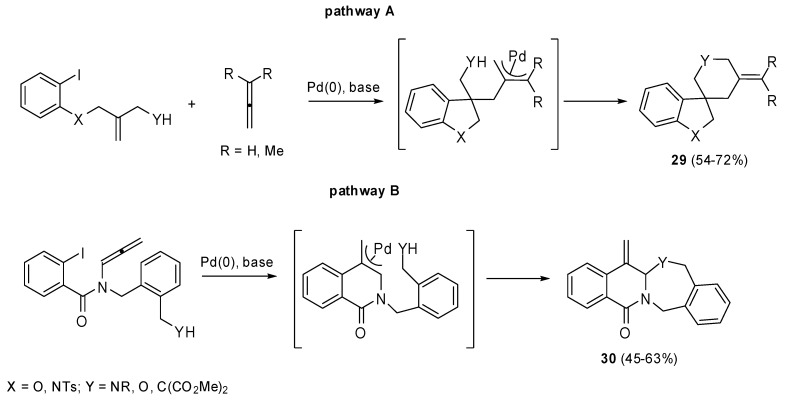
Synthesis of spiro- and fused heterocycles by Heck reaction.

Some palladium catalyzed carbo- and heteroannulation allene cascade reactions, which allowed the synthesis of spiro- and fused heterocycles, were proposed by the same group (Scheme 13) [20]. 

The oxidative process can be followed by an *exo-trig* cyclization, the intermolecular allene insertion and the intramolecular capture of the resultant (π-allyl)-palladium complex with a tethered nucleophile (pathway A, Scheme 13) affords the spiro-fused ring system **29**. Otherwise the (π-allyl)-palladium complex is generated by an *exo-dig* cyclization of the Ar-Pd species onto the adjacent 1,2-dienamide. The subsequent attack by a nucleophile permits the formation of bicyclic lactams **30** (pathway B, Scheme 13).

**Scheme 14:**
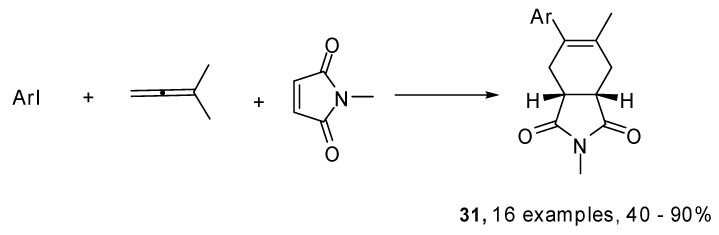
Three component Heck–Diels–Alder cascade process.

Moreover an intramolecular Heck reaction between aryl/heteroaryl iodides and alkylallenes was exploited to prepare functionalized 1,3-dienes (Scheme 14). These were subsequently reacted with various dienophiles to give the corresponding Diels–Alder adducts **31** [21].

Optimum reaction conditions were found to depend on the substituents present in the aryl iodide. It was necessary to work at 120 °C for 48 h to get good yields in the case of electron withdrawing groups, whereas the best results were obtained at 90 °C, when electron donating groups were present on the aromatic ring. This is possibly due to the reduced stability of the ArPdI complex.

**Scheme 15:**
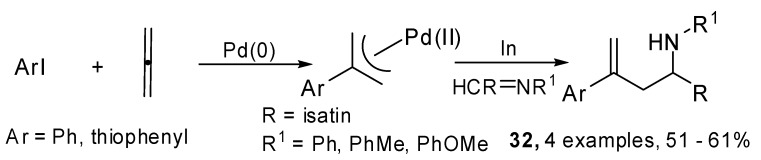
Cascade allylation of isatin imines.

A three component palladium-indium mediated diastereoselective cascade allylation of imines with allenes and aryl iodides was proposed by Grigg and coworkers too [22]. The synthesis provided *N*-tosyl and *N*-aryl homoallyl amines **32**. Furthermore the use of enantiomerically pure *N*-*tert*-butanesulfinyl imines afforded the desired products with excellent diastereoselectivity. Firstly isatin imines were explored (Scheme 15), in this case a spiro-oxindole was obtained.

**Scheme 16:**
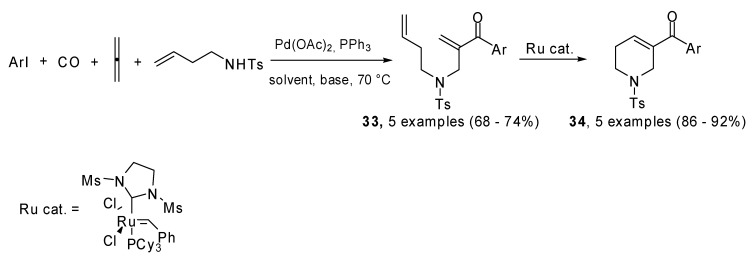
Four-component Pd(0) catalyzed cascade/ring closing metathesis.

1,6-Dienones and 1,7-dienones (**33**) were prepared using a four-component Pd(0) catalyzed four component process which involved carbon monoxide, allene and heteroaryl iodides. They generated a (π-allyl)-palladium species which reacted with alkene tethered nitrogen nucleophiles. A subsequent ring closing metathesis produced five- and six-membered *N*-heterocyclic enones **34** which are active dipolarophiles in 1,3-dipolar cycloaddition reactions (Scheme 16) [23]. 

**Scheme 17:**
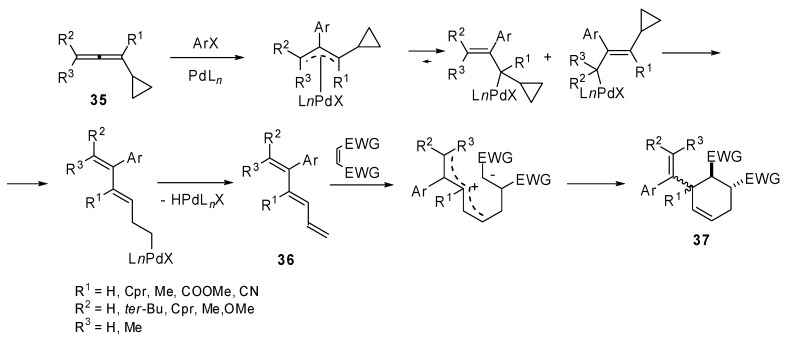
Domino Heck Diels-Alder reaction by substituted 1,3-dicyclopropyl-1,2-propadienes.

The original studies on the use of cyclopropylallenes in cascade reactions involving palladium catalyzed coupling were carried out by de Meijere *et al*. 1,3-Dicyclopropyl-1,2-propadiene (**35**) was coupled with several aryl iodides and bromides under palladium catalysis in the presence of a dienophile which underwent a domino Heck–Diels–Alder reaction. 3-(1’-Arylalkenyl)-substituted cyclohexenes **37** were achieved in moderate to good yields [24]. Then the methodology was extended to differently substituted cyclopropylallenes [25]. When the reaction was performed without the added dienophile, the intermediate coupling product **36 **was isolated and characterized, unfortunately it underwent polymerization within a few hours at room temperature. Moreover the [4 + 2] cycloaddition was found to proceed in a non-concerted fashion (Scheme 17).

**Scheme 18:**
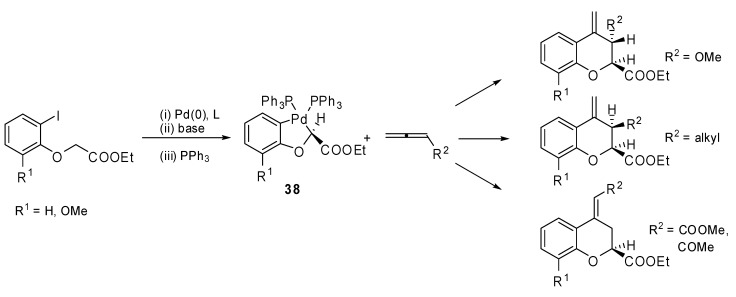
Synthesis of 2,3-disubstituted 3,4-dihydro-2*H*-1-benzopyrans.

**Scheme 19:**
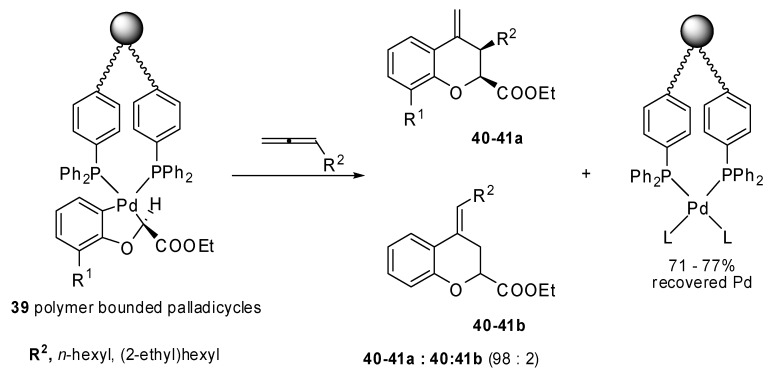
Synthesis of 2,3-disubstituted 3,4-dihydro-2*H*-1-benzopyrans using a polymer bounded palladicycle.

The insertion of monosubstituted allenes into stable oxapalladacycle **38** was studied by Malinakova and coworkers [26]. The proposed methodology allowed the synthesis of valuable 2,3-disubstituted 3,4-dihydro-2*H*-1-benzopyrans (Scheme 18), which could not be prepared by the classical palladium-catalyzed benzoannulation. Two adjacent stereogenic centers were generated and different, relevant benzopyrans were obtained with potential medicinal properties. The same reaction was applied to the polymer bound palladicycle **39** with remarkable results since more than 70% Pd was recovered (Scheme 19) [27]. 

**Scheme 20:**
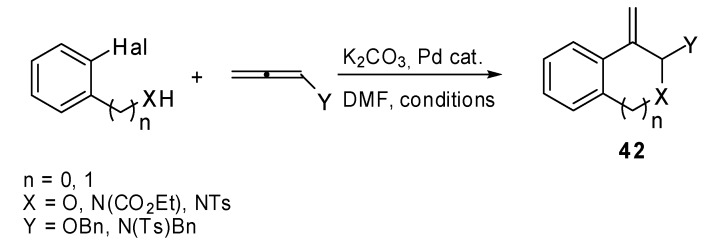
Annulation reaction of heteroatom-substituted allenes.

Recently a synthesis of condensed heterocycles **42** was proposed**.** It exploited the intramolecular Heck reaction applied to heteroatom substituted allenes and aryl halides (Scheme 20) [28]. The initially formed arylpalladium complex added to the allene and produced the corresponding π-allylpalladium intermediate, which readily underwent intramolecular nucleophilic attack by an oxygen or a nitrogen affording the annulated product **42**. Despite the presence of two possible attack positions, α or γ, only the α-site attack product was recovered regardless of the bulkiness of the allene substituent. There are two hypotheses to explain this selectivity. The electronegativity of the allene heteroatom renders the α position more electron positive than γ-position or the palladium complex is reductively eliminated with the assistance of the heteroatom and the cyclization proceeds without the participation of the palladium complex.

**Scheme 21:**
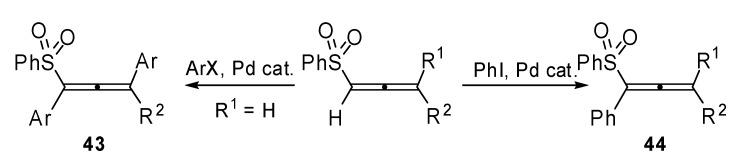
Heck type allenylation of aryl halides with allenes.

The first example of the Heck type allenylation of aryl halides with allenes was described by Ma and coworkers [29]. 1,3-Double arylated allenes **43 **were recovered when 3-monosubstituted 1,2-allenyl sulfones were used as reagents. Whereas the 1-monoarylation products **44** were obtained in the case of 3,3-disubstituted 1,2-allenyl sulfones (Scheme 21). The regioselectivity of the intermolecular carbopalladation shown, was completely opposite to what had been previously reported in literature. 

**Scheme 22:**
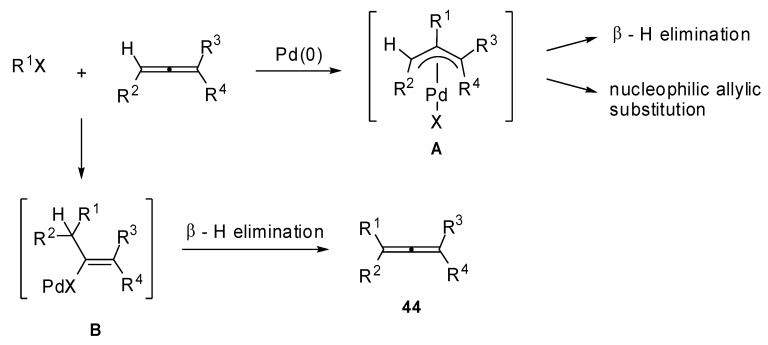
Proposed mechanism for the carbopalladation of allenes.

In fact, the carbopalladation of allenes normally affords a π-allylpalladium intermediate **A** (Scheme 22), which may undergo a β-hydride elimination or an allylic substitution affording nonallenic products. The formation of a vinylic palladium intermediate **B** is also reported in literature. The subsequent carboxylation leads to the formation of α,β-unsaturated alkenoates. Thus, the regioselectivity of the allene carbopalladation can be determined by the delocalization of the π-allylpalladium intermediate **A **and by the high energy/reactivity of the allene that will be formed by the β-H elimination of the vinylic intermediate **B**, as described in Scheme 22.

**Scheme 23:**
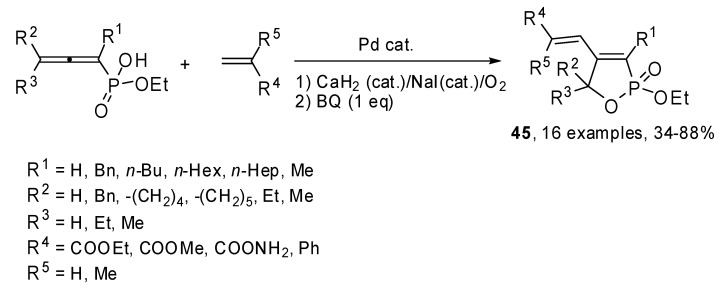
Cyclization-Heck reactions of monoesters of 1,2-allenyl phosphonic acids with allenes.

The same authors studied the cyclization−Heck reactions of monoesters of 1,2-allenyl phosphonic acids with allenes. As shown in Scheme 23, the reaction regio- and stereoselectively afforded 4-(1-*Z*-alkenyl)-2-ethoxy-2,5-dihydro[1,2]oxaphosphole 2-oxides **45**. Coupling were carried out in oxidative conditions, using CaH_2_(cat.)/NaI/O_2_ or benzoquinone to regenerate Pd(II) from the *in situ* formed Pd(0) [30]. 

**Scheme 24:**
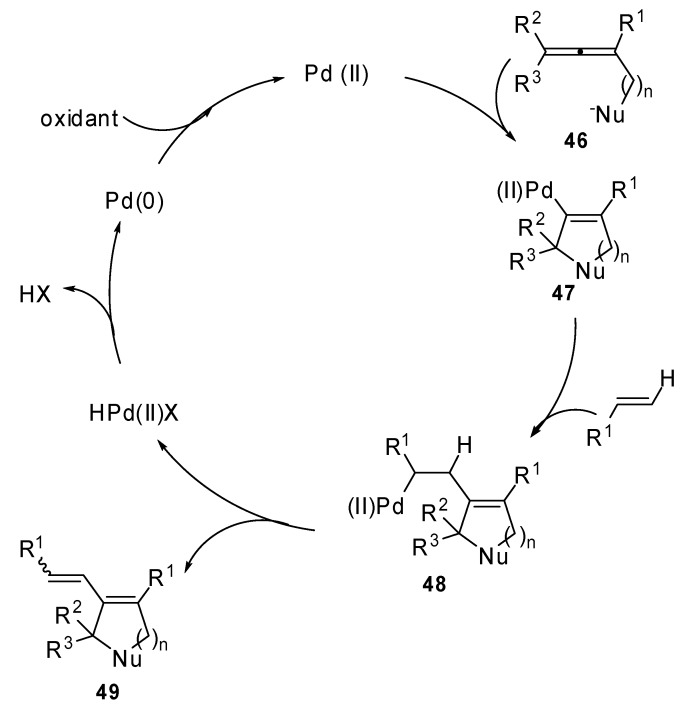
Proposed catalytic cycle for palladium mediated cyclization-Heck reaction.

The proposed mechanism is described in Scheme 24: an *endo*-mode cyclic nucleopalladation of allene **46** would form the cyclic palladium intermediate **47**. Then, the carbon-carbon alkene double bond is inserted into the C–Pd bond of **47** to form the complex **48.** This afforded the final product **49** after a β-H elimination.

**Scheme 25:**
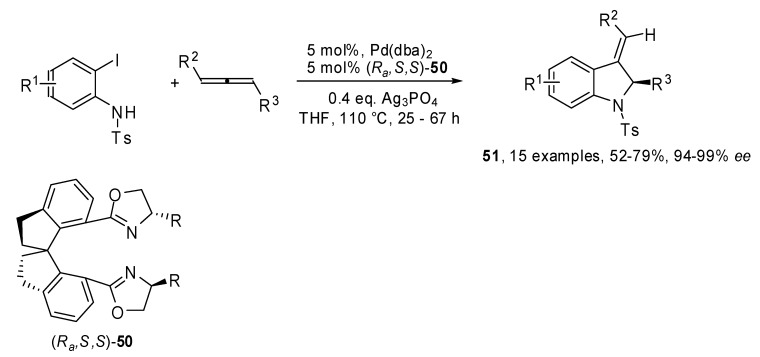
Enantioselective heteroannulations between allenes and 2-iodoanilines.

A new chiral spiro-bisoxazoline ligand β-naphtylmethyl-substituted spiro-BOX **50** was developed by the same group and applied to the enantioselective heteroannulations between allenes and 2-iodoanilines [31]. As shown in Scheme 25, the coupling produced 3-alkylideneindolines **51** in good yields and remarkable enantiomeric excesses.

A new synthesis of 2,3-disubstituted indole derivatives **54** was proposed by Sasaki *et al*. It was based on an intramolecular carbopalladation-anion capture cascade. Several *N*-(*o*-halophenyl)-allenamides **52 **were used as the starting reagents**. **Moreover, the introduction of an appropriate silicon group to the α-position of the allenamide, afforded the 2-silyl substituted indole derivatives, which are useful substrates for further Pd(0) catalyzed transformations at the C^2^ position. 

**Scheme 26:**
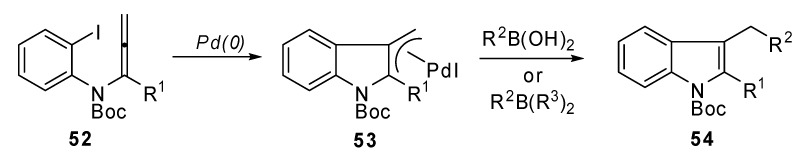
General strategy for the intramolecular carbopalladation-anion capture cascade of *N*-(*o*-halophenyl)allenamides.

**Scheme 27:**
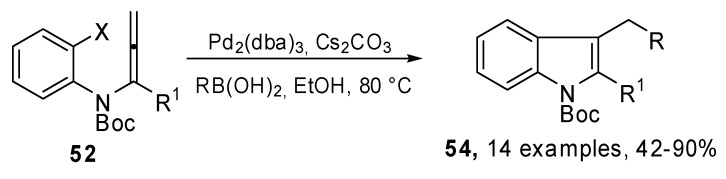
Synthesis of 3-substituted and 2,3- disubstituted indoles.

The general strategy, illustrated in Scheme 26, was based on the utilization of a *N*-(*o*-halophenyl)allenamide **52** which bore a substituent (R^1^) at the α-position of the allenamide. In this case a π-allylpalladium intermediate **53** could be generated *via* carbopalladation and could be trapped by a suitable nucleophile, such as an aryl or alkenyl boronic acid or alkylborane (Scheme 27) [32].

The methodology was then extended to the preparation of indoles-2,3-quinodimethanes which are highly reactive dienes that readily undergo Diels–Alder cycloaddition to get tetrahydrocarbazoles and related compounds [33].

**Scheme 28:**
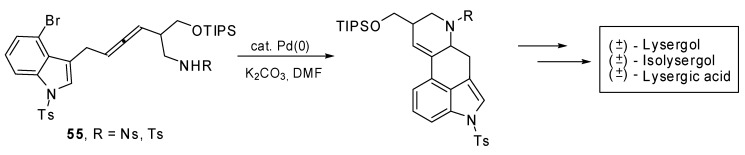
Synthesis of (±)-lysergic acid, (±)-lysergol and (±)-isolysergol by palladium catalyzed domino cyclization of amino allenes.

The palladium catalyzed domino cyclization of amino allenes **55 **was exploited to build the C/D ring system of ergot alkaloids. The total syntheses of (±)-lysergic acid, (±)-lysergol and (±)-isolysergol were achieved (Scheme 28) with this bisannulation as the key step [34]. 

The best reaction results in terms of yields and diastereoselectivity were obtained using Pd(PPh_3_)_4_ as the catalyst, K_2_CO_3_ as the base in DMF at 100 °C.

**Scheme 29:**
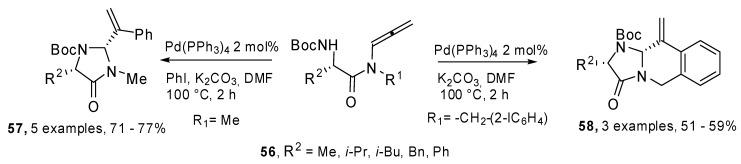
Heterocyclization reactions of allenamides by carbopalladation-intramolecular amination.

Allenamides of α-amino acids **56** were used for the preparation of enantiopure imidazolin-4-ones **57** and imidazoisoquinolinones **58** by means of a domino carbopalladation/allylic amination process. The authors also demonstrated the feasibility of the heterocyclization process with an amide group in the tether, without any interference of the carbonyl group (Scheme 29) [35]. 

**Scheme 30:**
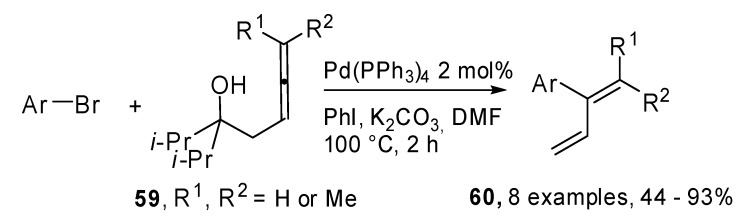
Synthesis of 2-aryl-1,3-alkadienes by aryl bromides and 3,4-alkadien-1-ols.

A new method for the synthesis of 2-aryl-1,3-alkadienes **60 **is illustrated in Scheme 30. Palladium-catalyzed 1-methylene-2-propenylation reactions of aryl bromides with 3,4-alkadien-1-ols **59** were exploited and a palladium mediated retro-allylation allowed the alcohols **59** to act as 1-methylene-2-propenyl metals. Moreover, they are inert in air and readily available [36].

**Scheme 31:**
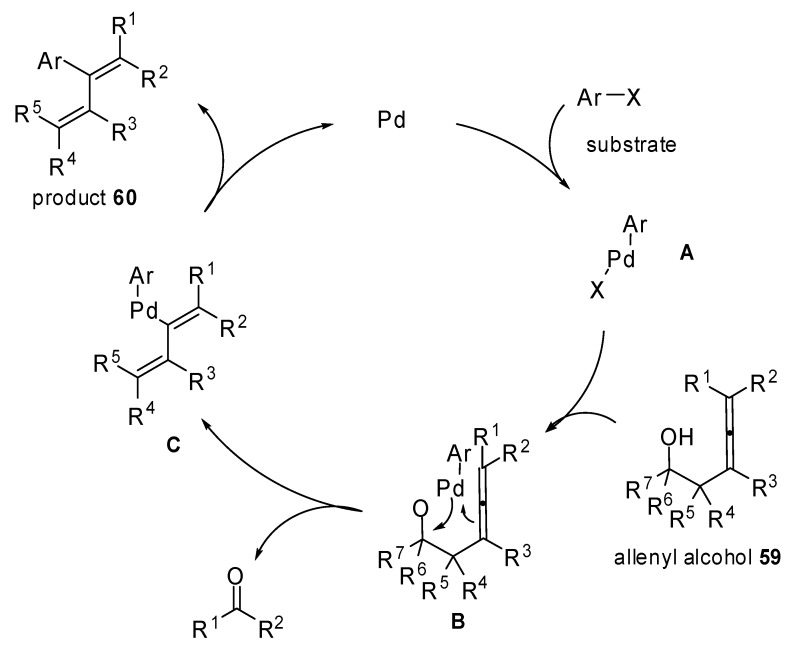
Hypothesized mechanism for the achievement of 2-aryl-1,3-alkadienes.

The mechanism hypothesized by the authors is reported in Scheme 31. The palladium alcoxide **B** is formed after the oxidative addition and the ligand exchange. Then a C–C bond cleavage proceeds via a six-membered cyclic transition state selectively providing the intermediate **C**. Finally the reductive elimination will furnish the desired 2-aryl-1,3-alkadienes **60**.

**Scheme 32:**
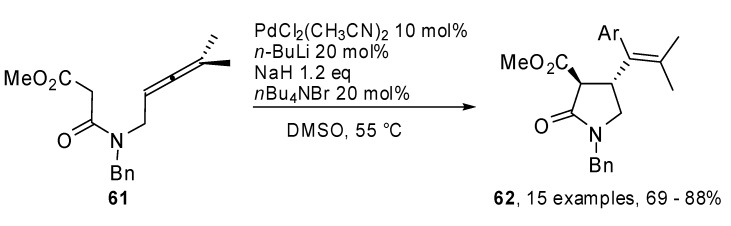
Synthesis of 4-(α-styryl) γ-lactams by β-aminoallene.

**Figure 1:**
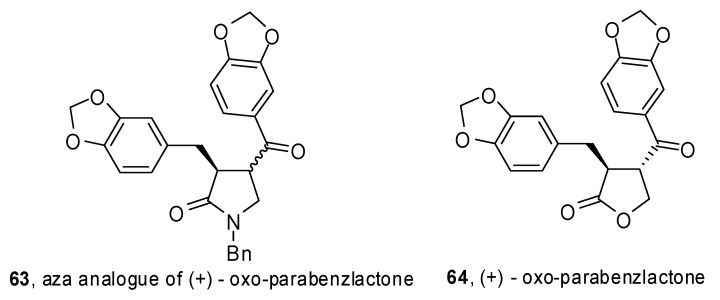
Aza analogue of oxo-parabenzlactone.

A phosphine-free Pd-catalyzed allene carbopalladation/allylic alkylation sequence was exploited by Poli and coworkers to get 4-(α-styryl) γ-lactams **62** from β-aminoallene **61 **(Scheme 32) [37]. High yields were obtained for electron-rich as well as electron-poor aryl iodides. Furthermore the reaction was completely regio- and stereoselective *versus* the 5-*exo trans* product. The synthetic sequence was extended to the preparation of an aza analogue of (+) oxo-parabenzlactone (**63**,Figure 1), a naturally occurring lignin.

**Scheme 33:**
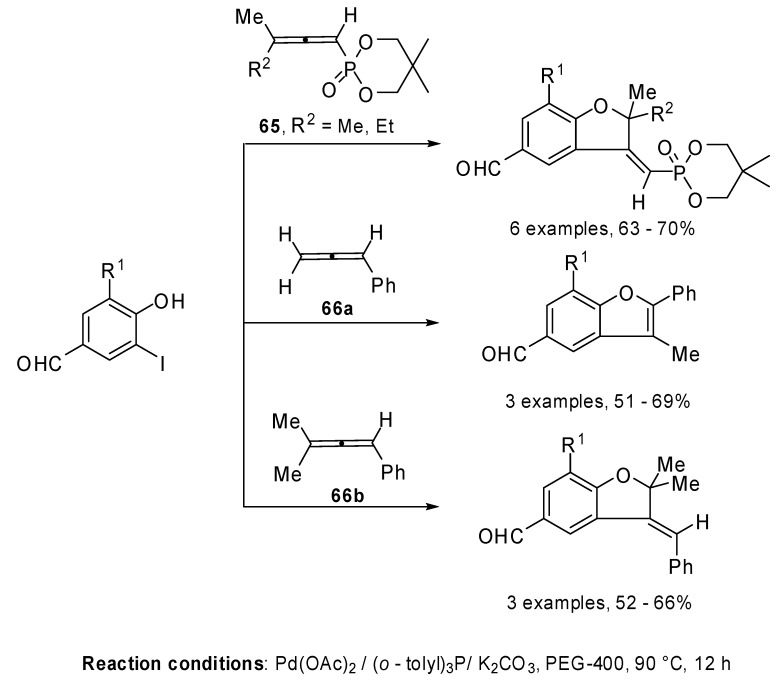
Reactions of allenylphosphonates and phenylallenes with functionalized iodophenols, 2-iodobenzoic acid in PEG 400.

The regioselective palladium catalyzed coupling reaction of allenylphosphonates **65** and phenylallenes **66 **with functionalized iodophenols, 2-iodobenzoic acid and 2-iodobenzyl alcohol was investigated [38]. Recently a similar reactivity stydy using PEG 400 as the solvent was described. The reaction allowed the regioselective formation of aldehyde-functionalized benzofuranes and benzopyrans. The above mentioned results demonstrated that a [β,γ] attack on the allene was preferred when benzofurans or benzopyrans were formed, except in the case of PhC=C=CCH_2_ (**66a**) where a [β,α] attack was observed (Scheme 33) [39].

## 4. Conclusions

In conclusion, this review describes some important uses of the Mizoroki–Heck coupling, reported in the literature during the last ten years, with particular regards to 1,2- and 1,3-dienes. In general both these systems form π-allyl Pd intermediates in Pd(0) coupling and show a particular chemical behavior. Some examples of the application of the Heck reaction to 1,3-dienes are also reported. Moreover, some examples of the Mizoroki−Heck coupling of 1,2-dienes are reported in which the reaction is a key step in the synthesis of biologically relevant molecules. Furthermore, particular reactivities of allenes in the Mizoroki-Heck reaction are described, such as some couplings in which a vinylic Pd-intermediate is formed or a Pd-In mediated transformation is involved.
